# Preserving Quality and Extending Shelf Life of Climacteric Persimmon Fruits Using Melatonin and Modified Atmosphere Packaging

**DOI:** 10.1002/fsn3.70143

**Published:** 2025-04-09

**Authors:** Emine Kucuker, Muttalip Gundogdu, Emrah Güler, Ahmet Sumbul, Onur Tekin, Bulent Hallac

**Affiliations:** ^1^ Department of Horticulture, Faculty of Agriculture Siirt University Siirt Türkiye; ^2^ Department of Horticulture, Faculty of Agriculture Bolu Abant İzzet Baysal University Bolu Türkiye; ^3^ Department of Plant and Animal Production, Suşehri Timur Karabal Vocational School Sivas Cumhuriyet University Sivas Türkiye; ^4^ Department of Horticulture, Faculty of Agriculture Van Yuzuncu Yil University Van Türkiye; ^5^ Department of Food Engineering, Faculty of Engineering Siirt University Siirt Türkiye

**Keywords:** *Diospyros kaki*, melatonin, modified atmosphere packaging, organic acids

## Abstract

Quality and product losses during the postharvest storage of climacteric persimmon fruits pose significant challenges due to their short shelf life and limited marketing period. This study examined the effects of melatonin (1 mM) and modified atmosphere packaging (MAP) on preserving the quality, biochemical properties, and organic acid contents of persimmons, as well as extending their shelf life during storage at 0°C ± 0.5°C. The MAP resulted in the lowest weight loss, recording only 6.63%. The combination of melatonin and MAP provided the highest fruit firmness at 8.69 kg/cm^2^, along with a total antioxidant level of 12.40 μmol TE 100 g^−1^. Additionally, this treatment improved total phenolic content and most individual organic acids, except for fumaric acid, which was highest in the MAP treatment. The organic acid contents of the fruits varied during storage, depending on the specific acid. Malic acid was the predominant organic acid and was consistently better preserved by the treatments compared to the control. It is important to note that the reduction in malic acid was approximately 1.5 times less in the treated fruits than in the controls, while the decline of other organic acids was 3–4 times greater. In conclusion, both MAP and the combination of melatonin and MAP were effective methods for preserving the quality attributes and extending the shelf life of persimmon fruits.

## Graphical abstract

Melatonin (1 mM) and MAP effectively preserved fruit color (*L*, *a*, *b*) and firmness while reducing weight loss. The combination of Mel + MAP maintained higher antioxidant content and better organic acid retention, particularly malic acid. While control fruits showed significant declines in color and organic acids over 28 days, treated fruits retained quality longer. Succinic and tartaric acids increased over time, with the highest succinic acid levels in Mel + MAP. These treatments significantly extended persimmon shelf life.

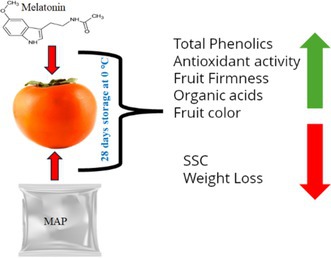

## 1 Introduction

Persimmon ( *Diospyros kaki* ) is a source of important fiber as well as having a unique flavor and containing many bioactive compounds including polyphenols, flavonoids, minerals, carotenoids, steroids, and terpenoids (Pérez‐Burillo et al. 2018). Climacteric persimmon fruits are highly sensitive to ethylene, which accelerates the ripening process (Harima et al. 2003). Therefore, inhibiting ethylene biosynthesis and its effects can slow down the ripening process and extend the storage life (Luo 2007). Generally, storing fresh fruits at low temperatures is effective in maintaining quality. Low temperatures can suppress ethylene production and other physiological activities. However, persimmon fruits are sensitive to storage temperatures (Besada et al. 2008), and chilling injury occurs at low temperatures (Orihuel‐Iranzo et al. 2010; Niazi et al. 2021). Chilling stress during cold storage typically leads to the accumulation of reactive oxygen species (ROS) in persimmon fruits, which ultimately causes numerous physiological disorders, such as fruit softening, browning, skin color changes, and loss of antioxidant compounds (Besada et al. 2014; Saleem et al. 2020).

In recent years, various postharvest treatments have been utilized to mitigate chilling injury and maintain the quality of persimmon fruits during cold storage, including methyl jasmonate (Bagheri and Esna‐Ashari 2022), combinations of hot water treatment and calcium lactate (Naser et al. 2018), hydrogen sulfide and γ‐aminobutyric acid (Niazi et al. 2021), combinations of controlled atmosphere and edible coatings (Sortino et al. 2022), ethanol and 1‐methylcyclopropene (1‐MCP) combinations (Tessmer et al. 2019), CO_2_ and 1‐MCP combinations (Zhang et al. 2018a; Min et al. 2018), modified atmosphere packaging (MAP) (Karaca and Kurşun 2019), 1‐MCP and aminoethoxyvinylglycine (AVG) combinations (Win et al. 2018), oxalic acid and 1‐MCP (Li et al. 2018), melatonin and 1‐MCP (Jiao et al. 2022a), and MAP (Mangaraj and Goswami 2011).

Recent studies have shown that combining different treatments used in the cold storage of persimmons is more effective in maintaining postharvest quality. This may be due to the combined physiological regulatory effects of these treatments (Jiao et al. 2022a). However, there is limited information on the effects of MAP and melatonin applications on the cold storage performance of persimmons. In light of the literature, this study hypothesized that MAP and melatonin treatments could preserve the fruit quality and biochemical content of persimmons during cold storage.

## 2 Materials and Methods

The material of the study consisted of the Rojo Brillant persimmon variety. The fruits were harvested at the firm‐ripe stage using scissors, leaving their caps intact. Fruits with similar shape, size, and color characteristics were placed in perforated bags and transported to the laboratory. The fruits were divided into four groups, each consisting of three replicates, with 150 fruits per replicate. The first group consisted of control, the second group consisted of 1 mM melatonin, the third group consisted of MAP, and the fourth group consisted of a combination of melatonin + MAP application. In the control group, the fruits were only washed with distilled water without any further treatment. The second group consisted of fruits immersed in a melatonin solution for 15 min. The third group included fruits stored in modified atmosphere packaging that is polyethylene (PE) and has an O_2_ permeability of 150–300 cc m^2^ day, CO_2_ permeability of 300–800 cc m^2^ day, and a water vapor transmission rate (WVTR) of 0.5–1.5 g m^2^ day. The fourth group consisted of fruits first immersed in a melatonin solution for 15 min, air‐dried under room conditions for 20 min, and then stored in modified atmosphere packaging. Each package consisted of 10 fruits, and the atmosphere composition in the bags was 3%–5% O_2_, 5%–10% CO_2_, and balance nitrogen. Throughout the study, all fruits were stored at 0°C ± 0.5°C with 90%–95% relative humidity for 28 days. The quality attributes and biochemical contents of the fruits were assessed on Days 0, 7, 14, 21, and 28.

### 2.1 Weight Loss, Fruit Flesh Firmness, Soluble Solids Content, Titratable Acidity, and Color Properties

After determining the initial weights of persimmon fruits, subsequent weights were recorded on the 7th, 14th, 21st, and 28th days. Weight losses were then calculated as percentages. To measure fruit flesh firmness, the peel was removed from the center of 10 fruits in two directions using a penetrometer (FT‐327; McCormick, WA, USA), and firmness was recorded in kg/cm^2^. The soluble solid content (SSC) of the juice extracted from the fruits was determined as a percentage using a digital refractometer (Atago PAL‐1, Japan), and the titratable acidity of the fruits was measured as percentages of malic acid via the titration method that final pH was adjusted to 8.1 by adding 0.1 N NaOH. The skin color values of the persimmon fruit (L*, a*, b*) were measured from two different sides of five fruit samples using a colorimeter (3nh NR60C, China). The average of the two readings from each side was calculated to determine the final color values.

### 2.2 Total Phenolic Content and Total Antioxidant Activity

For each replicate, 10 fruits were homogenized, and approximately 30 mL of homogeneous fruit sample was centrifuged at 12000 rpm for 35 min in a centrifuge set at 4°C. Total phenolic content and total antioxidant activity were determined using the supernatant with the help of a UV–Vis spectrometer (Shimadzu, Kyoto, Japan). Total phenolic content was determined as gallic acid as mg GAE/100 g^−1^ according to the method specified by (Singleton and Rossi 1965), and total antioxidant activity was determined as μmol 100 g^−1^ in Trolox equivalent (TE) according to the method specified by (Benzie and Strain 1996).

### 2.3 Determination of Organic Acids

Five grams of the fruit sample from a mixture of 10 fruits of each sample were homogenized with 0.009 N H_2_SO_4_ in a 1:1 ratio and rotated at 15000 rpm for 15 min. The supernatant obtained was filtered through a 0.45 μm membrane filter and then passed through a SEP‐PAK C18 cartridge. Organic acids were analyzed using an Agilent HPLC 1100 series G1322A (Germany) equipped with an Aminex HPX‐87H column (300 × 7.8 mm, Bio‐Rad Laboratories, Richmond, CA, USA) (Bevilacqua and Califano 1989).

### 2.4 Statistical Analysis

Data was analyzed using a two‐way ANOVA through the statistical software SAS 9.1 (SAS Institute Inc., Cary, NC, USA). When the F test indicated a significant result, means were compared using the Tukey's HSD (honestly significant difference) test. Pearson's pairwise correlations were calculated using the “corrplot” package in R Studio version 2022.12.0 (Wei et al. 2017). Principal component analysis (PCA) was performed with JMP 16 (SAS, USA) to examine the interactions between melatonin and MAP treatments, as well as the quality and biochemical properties of the fruits. Additionally, data visualization included heatmap analysis using the “Bioconductor” package in R (Gentleman et al. 2004).

## 3 Results and Discussion

**FIGURE 1:**
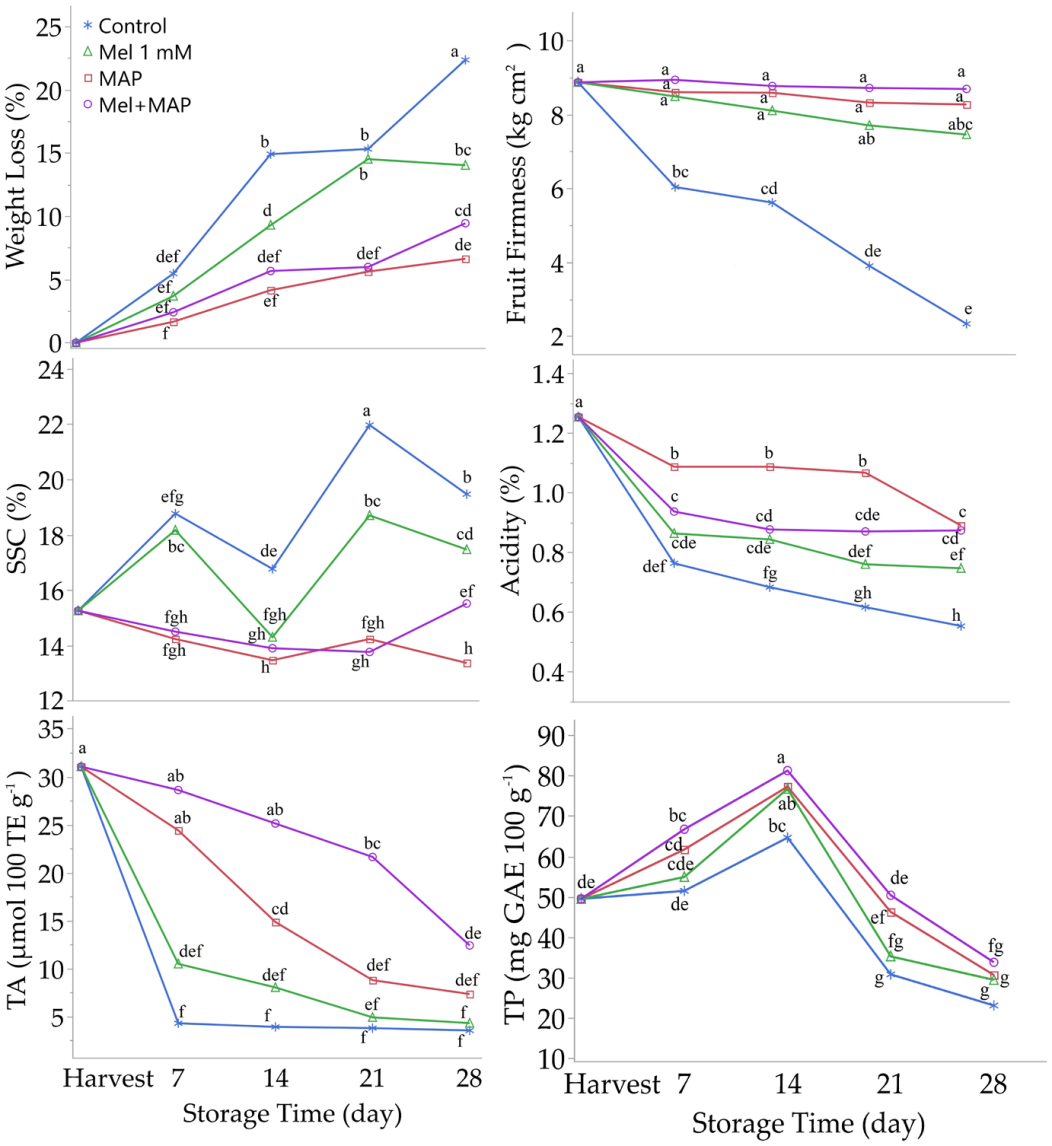
Effect of post‐harvest Melatonin and MAP applications on quality traits of fruits. Different letters on top of the storage periods indicate significant differences at *p* ≤ 0.05.

Weight loss is one of the most important factors determining the economic value of fruits during storage. In this study, it was observed that weight loss increased over the storage period, with the highest weight loss (13.11%) occurring on the 28th day of storage. On Days 7, 14, 21, and 28, the lowest weight loss was recorded in the MAP application (1.66%, 4.15%, 5.62%, and 6.63%, respectively). The melatonin + MAP treatment showed a positive effect in reducing weight loss compared to the melatonin treatment alone (Figure 1, Table S1). Respiration and transpiration processes during storage are the primary causes of weight loss in fruits (González‐Aguilar et al. 2009), and weight loss increases proportionally with the storage duration (Ozturk et al. 2021). In this study, MAP applications significantly delayed weight loss. By altering the gas concentration around the fruit, MAP delays the breakdown of cell walls, reduces respiration (Wang et al. 2019), and minimizes water loss (Ozturk et al. 2020), thereby mitigating weight loss. MAP has been shown to be effective in reducing weight loss in many fruit types (Aglar et al. 2017; Candir et al. 2018; Yarılgaç et al. 2022; Avcı et al. 2023; Ogurlu et al. 2024). Melatonin reduces deterioration and losses by delaying the activity of enzymes in fruits (Zhang et al. 2018b). It helps maintain cell wall integrity and inactivates enzymes involved in ROS metabolism, which are particularly effective during cell senescence (Küçüker et al. 2024). However, the effectiveness of melatonin can vary depending on the fruit type, storage conditions, application concentration, and duration (Jiao et al. 2022b).

Fruit firmness generally decreased during the storage period. As storage time increased, melatonin and MAP applications statistically significantly preserved fruit firmness (*F* Storage time × Melatonin and MAP: 7.72; *p* ≤ 0.001), with the melatonin + MAP treatment showing the highest effect. The reduction in fruit firmness is proportional to the increase in ethylene synthesis (Figure 1, Table S1). Ethylene reduces fruit firmness by enhancing the activity of enzymes that hydrolyze the cell wall (Khan et al. 2008). MAP applications slow down the loss of fruit firmness by reducing the degradation of cellulose and pectin in the cell wall (Azene et al. 2014) and by lowering O_2_ concentrations during storage, thereby reducing ethylene synthesis (Khan et al. 2008). Melatonin may play a role in maintaining fruit firmness by inhibiting the activity of enzymes responsible for softening and preventing the breakdown of the cell wall structure (Kucuker et al. 2023). The finding that the combination of melatonin and MAP application was the most effective method in maintaining fruit firmness is consistent with the literature. Many researchers have reported that MAP and melatonin applications are effective methods for preserving fruit firmness during storage (Aglar et al. 2017; Yarılgaç et al. 2022; Ogurlu et al. 2024; Küçüker et al. 2024; Kucuker et al. 2023, 2024).

With fruit ripening, SSC increases. In this study, the SSC of the fruits increased on Days 7 and 21 but decreased on Days 14 and 28. Throughout the storage period, SSC levels showed inconsistent changes depending on the treatments. While the melatonin treatment caused fluctuations in SSC, MAP maintained lower SSC values during storage. At the end of the storage period, the melatonin treatment increased SSC by approximately 15%, whereas the MAP treatment decreased it by around 12%. The acid content of the fruits decreased by approximately 38% during the storage period. MAP treatments slowed the reduction in acidity. At the end of the storage period, the decrease in acidity was approximately 40% with the melatonin treatment and about 29% with the MAP treatment (Figure 1, Table S1). SSC and acidity, which are indicators of harvest maturity, are crucial fruit quality attributes that determine the fruit's quality and consumer acceptability. As ripening progresses, the hydrolysis of insoluble polysaccharides increases SSC, while acidity decreases (Abd El‐Gawad et al. 2019). The inconsistent effects of the treatments on SSC observed in this study have also been reported in the literature for various fruit types. Studies on pears (Ogurlu et al. 2024), figs (Kucuker et al. 2023), hawthorn (Küçüker et al. 2024), and strawberries (Liu et al. 2018) have documented inconsistent changes in SSC during storage. The fluctuations in SSC content during cold storage can be attributed to processes such as the conversion of sugars into CO_2_ and H_2_O due to increased respiration rates, the conversion of starch to sugars, the increase in dry matter content due to water loss, and the breakdown of polysaccharides in the cell wall (Díaz‐ Mula et al. 2012; Vieira et al. 2016). The reduction in acidity during storage is likely due to the consumption of organic acids in respiration, which increases with higher respiration rates (Archana and Suresh 2019).

The storage duration and treatments were found to have a statistically significant effect on the total antioxidant activities of persimmon fruits (*F* Storage time: 51.10; *F* Storage time × Melatonin and MAP: 12.60; *p* ≤ 0.001). During storage, total antioxidant activity showed a decreasing trend, with a reduction of approximately 78%. The highest decrease in total antioxidant activity was observed in the control treatment, while the melatonin + MAP combination was the most effective in slowing this reduction. By the end of the storage period, the decrease in total antioxidant activity was approximately 89% in the control treatment and 60% in the melatonin + MAP treatment. The storage duration and treatments also had a statistically significant effect on the total phenolic content of persimmon fruits (*F* Storage time: 3.89; *F* Storage time × Melatonin and MAP: 17.28; *p* ≤ 0.001), although this effect was inconsistent. Total phenolic content increased until Day 14 of storage and then decreased in the subsequent days. On Day 14, the highest total phenolic content (approximately 65%) was observed with the melatonin + MAP combination. By the end of the storage period, the smallest reduction in total phenolic content (approximately 32%) was also observed with the melatonin + MAP treatment (Figure 1, Table S1). MAP treatments slow the loss of total phenolic content and total antioxidant activity by altering the gas composition around the fruits, thereby reducing respiration rates (Azene et al. 2014; Mohebbi et al. 2015). Melatonin, on the other hand, is known to increase the amount of total phenolic compounds during storage by activating the phenylalanine pathway, which promotes the production of anthocyanins and phenolic compounds (Pang et al. 2020). The increase in total phenolic content during storage can be attributed to anthocyanin accumulation, while the decrease is likely due to enzymatic activity that degrades phenolic compounds (Fawole and Opara 2013). Consistent with the findings of this study, the positive effects of MAP and melatonin treatments on total phenolic content and total antioxidant activity have been reported in studies on various fruit species (Ozturk et al. 2020; Aglar et al. 2017; Yarılgaç et al. 2022; Kucuker et al. 2023).

The skin color values (L*, a*, and b*) of the fruits exhibited a decreasing trend with respect to storage duration and treatments. In the control treatment, the L* value of the fruits was higher compared to treated fruits. MAP treatment increased the L* value, indicating brightness, on Days 7 and 14, while it decreased on Days 21 and 28. Overall, MAP slowed the reduction in L* values compared to other treatments. The a* value, representing redness, displayed inconsistent changes according to storage duration and treatments. Melatonin treatment showed an increase in a* values on Days 7 and 21, and a decrease on Days 14 and 28, compared to the control. However, melatonin was effective in maintaining the a* color value of the fruit skin compared to other treatments. Similarly, the b* value, also indicative of redness, exhibited inconsistent changes with storage duration and treatments. MAP treatment increased the b* value on Days 7 and 14 but showed a decreasing trend on Days 21 and 28 compared to the control. MAP was effective in maintaining the b* color value, outperforming other treatments (Table 1). Climacteric persimmon, being a fruit sensitive to cold, could benefit from ethylene inhibition as a vital protective mechanism against cold stress, potentially slowing the process of quality deterioration (Jiao et al. 2022b). Ethylene production in fruits can lead to changes in skin color by increasing the activity of hydrolyzing enzymes, enhancing respiration intensity, and inducing the degradation of chlorophyll and carotenoid pigments (Rasouli and Khademi 2018). A positive relationship exists between ethylene production and color changes in fruits (Díaz‐ Mula et al. 2011). Ethylene production can be slowed in cold environments with varying atmospheres, thereby delaying fruit color development (Argenta et al. 2003). In this study, the positive effects of MAP and melatonin treatments on fruit skin color values can be attributed to their ability to reduce ethylene production, thereby preventing ripening and associated color changes.

**TABLE 1:** Effect of melatonin and MAP treatments on color values of persimmon fruits during storage.

Storage time	*L*	*a*	*b*
Harvest	40.90 ± 0.17a	2.32 ± 0.08a‐c	38.95 ± 0.29a
7th day	33.75 ± 0.99b	3.79 ± 0.28a	33.40 ± 1.04a
14th day	25.76 ± 1.16c	2.39 ± 0.43bc	25.44 ± 1.13b
21th day	16.47 ± 0.86d	3.12 ± 0.49ab	14.96 ± 1.23c
28th day	15.69 ± 1.72d	1.49 ± 0.54c	15.08 ± 2.18c
*Storage time × Melatonin and MAP interaction*			
Harvest	40.90 ± 0.17a	2.32 ± 0.08b‐e	38.95 ± 0.29a
7th day			
Control	32.86 ± 1.13 cd	3.77 ± 0.57a‐c	31.25 ± 0.88b
Mel1mM	29.25 ± 0.48d	4.57 ± 0.43a	29.17 ± 0.50bc
MAP	37.45 ± 0.31ab	2.97 ± 0.39a‐d	36.41 ± 0.36a
Mel + MAP	35.43 ± 1.01bc	3.86 ± 0.65a‐c	36.77 ± 1.03a
14th day			
Control	23.65 ± 0.99e	2.05 ± 1.47c‐e	22.38 ± 1.24de
Mel 1 mM	20.88 ± 0.35e‐g	1.40 ± 0.47de	21.51 ± 0.70d‐f
MAP	29.51 ± 0.75d	3.82 ± 0.12a‐c	29.09 ± 0.12bc
Mel + MAP	28.99 ± 1.26d	2.30 ± 0.43b‐e	28.76 ± 1.20bc
21th day			
Control	18.20 ± 0.72 g‐ı	4.19 ± 0.61ab	16.97 ± 2.17f‐h
Mel 1 mM	19.18 ± 2.33f‐h	4.34 ± 1.29a	18.57 ± 3.35e‐g
MAP	14.24 ± 0.46ıj	1.93 ± 0.42c‐e	12.33 ± 0.45hı
Mel + MAP	14.25 ± 0.36ı	2.01 ± 0.58c‐e	11.97 ± 0.70hı
28th day			
Control	22.63 ± 4.80ef	4.05 ± 1.09ab	24.39 ± 5.04 cd
Mel 1 mM	10.01 ± 0.81j	1.10 ± 0.70de	7.35 ± 1.61ı
MAP	15.39 ± 0.35hı	0.44 ± 0.41e	14.49 ± 0.88gh
Mel + MAP	14.71 ± 0.94ı	0.35 ± 0.17e	14.11 ± 1.32 gh
ANOVA			
*F* Storage time	37.07***	35.82***	0.45^ns^
*F* Storage time × Melatonin and MAP	39.55***	4.02***	29.99***

*Note:* Different letters in the same column indicates statistical differences at *p* ≤ 0.05.

Abbreviation: ns, not significant.

*** *p* ≤ 0.001.

Tartaric acid was the most abundant organic acid in persimmon fruits, followed by malic acid and citric acid, while oxalic acid had the lowest concentration. Organic acid levels showed inconsistent changes depending on storage duration and treatments. Melatonin + MAP treatment increased the oxalic acid content compared to the control and other treatments throughout storage. After Day 21, a decreasing trend in oxalic acid content was observed, with a reduction compared to the initial content at the end of storage. MAP treatment positively affected citric acid content up to the end of Day 21, but all treatments showed a reduction in citric acid at the end of storage. Melatonin + MAP treatment preserved malic acid content during storage. Succinic acid content increased throughout storage, with different impacts from treatments. At the end of storage, the highest succinic acid content was observed with melatonin + MAP treatment. Although fumaric acid content increased on Day 7, it generally declined throughout storage. MAP treatment was effective in maintaining fumaric acid content at the end of storage. Tartaric acid content showed an increasing trend up to Day 21, followed by a decreasing trend. Melatonin + MAP treatment positively influenced tartaric acid content during storage (Table 2). The content and concentration of organic acids, which are important quality parameters in fruits, can vary according to fruit type and variety, and their levels decrease as fruit maturity increases (Kıralan and Gündoğdu 2021). Generally, MAP (Ogurlu et al. 2024) and melatonin treatments (Küçüker et al. 2024; Kucuker et al. 2023; Tijero et al. 2019; Liu et al. 2020) have been shown to have significant effects in preserving the organic acid content in fruits. However, the impact of treatments on organic acid changes varies depending on the type of organic acid and storage duration. Melatonin, for example, has been known to reduce the activity of CAD and POD enzymes, which leads to increased lignin accumulation, thereby increasing the amounts of organic acids such as tartaric, malic, and oxalic acids (Wang et al. 2021).

**TABLE 2:** Effect of melatonin and MAP treatments on organic acid contents of persimmon fruits during storage (mg/100 g).

Storage time	Oxalic	Citric	Malic	Succinic	Fumaric	Tartaric
Harvest	4.28 ± 0.08bc	43.34 ± 0.62a	561.26 ± 3.37a	8.81 ± 0.08bc	11.56 ± 0.06b	220.57 ± 5.64 cd
7th day	6.44 ± 0.35a	36.45 ± 2.79a	493.98 ± 24.32a	11.82 ± 1.52c	25.79 ± 4.37a	649.89 ± 131.93bc
14th day	4.94 ± 0.36b	35.77 ± 4.11a	514.86 ± 18.73a	14.10 ± 2.44bc	15.70 ± 2.38b	815.20 ± 121.55b
21th day	3.25 ± 0.36c	30.76 ± 3.88a	480.82 ± 25.54ab	26.51 ± 5.26b	14.01 ± 1.31b	1217.15 ± 174.95a
28th day	1.93 ± 0.26d	3.17 ± 0.73b	413.02 ± 32.01b	47.02 ± 6.69a	8.87 ± 1.67b	291.97 ± 48.25d
*Storage time × Melatonin and MAP interaction*						
Harvest	4.28 ± 0.08d‐f	43.34 ± 0.62a‐c	561.26 ± 3.37a‐d	8.81 ± 0.08f‐h	11.56 ± 0.06d‐g	220.57 ± 5.64de
7th day						
Control	6.28 ± 0.13ab	26.10 ± 1.26de	379.49 ± 30.32 gh	9.55 ± 2.68f‐h	11.25 ± 0.33e‐g	173.73 ± 66.62e
Mel1mM	5.53 ± 0.38b‐d	38.43 ± 8.10bc	535.67 ± 0.00a‐f	10.61 ± 3.88f‐h	28.30 ± 3.37ab	977.69 ± 114.45bc
MAP	6.20 ± 0.34a‐c	46.18 ± 0.25ab	476.00 ± 19.52c‐g	12.71 ± 4.64f‐h	25.40 ± 1.02bc	303.07 ± 67.19de
Mel+MAP	7.77 ± 1.06a	35.09 ± 0.08 cd	584.77 ± 3.10ab	14.41 ± 0.02f‐h	38.19 ± 14.88a	1145.09 ± 104.99b
14th day						
Control	4.49 ± 0.65d‐f	22.42 ± 3.65e	434.55 ± 18.59 fg	4.89 ± 1.54gh	4.34 ± 0.10 g	477.96 ± 135.33de
Mel1mM	4.26 ± 0.81d‐f	34.71 ± 9.23 cd	547.15 ± 11.70a‐e	13.41 ± 0.46f‐h	13.96 ± 0.79c‐g	1049.11 ± 20.53bc
MAP	4.66 ± 0.15c‐e	52.68 ± 0.00a	485.74 ± 8.59a‐g	23.05 ± 1.67e‐g	21.27 ± 0.70b‐e	491.70 ± 26.47de
Mel+MAP	6.38 ± 0.57ab	33.28 ± 6.19 cd	592.02 ± 4.14a	15.02 ± 6.50e‐h	23.22 ± 3.73b‐d	1242.02 ± 279.20b
21th day						
Control	2.28 ± 0.12 g‐ı	15.98 ± 1.35ef	403.21 ± 75.71gh	2.16 ± 0.42 h	7.95 ± 0.35 fg	667.66 ± 185.68 cd
Mel1mM	3.15 ± 0.90e‐h	22.60 ± 3.45e	463.28 ± 21.41d‐g	32.98 ± 11.30c‐e	12.37 ± 0.28d‐g	1245.82 ± 182.16b
MAP	3.52 ± 1.04e‐g	48.70 ± 0.26ab	476.33 ± 0.00b‐g	44.21 ± 0.00b‐d	18.23 ± 0.94b‐f	1061.13 ± 0.00bc
Mel+MAP	4.04 ± 0.45d‐f	35.76 ± 1.33 cd	580.48 ± 4.34a‐c	26.68 ± 2.46d‐f	17.49 ± 1.48b‐f	1894.00 ± 461.65a
28th day						
Control	0.90 ± 0.34ı	1.34 ± 0.38 g	315.19 ± 112.69 h	22.74 ± 4.03e‐g	3.14 ± 0.32 g	75.32 ± 27.66e
Mel	2.29 ± 0.16 g‐ı	1.75 ± 0.02 g	447.53 ± 52.69e‐g	52.55 ± 21.45ab	10.07 ± 5.61e‐g	233.64 ± 20.29de
MAP	1.65 ± 0.27hı	7.26 ± 0.40 fg	440.49 ± 21.85e‐g	45.95 ± 0.15bc	12.66 ± 1.29d‐g	360.80 ± 33.46de
Mel+MAP	2.89 ± 0.40f‐h	2.33 ± 0.16 g	448.87 ± 13.48e‐g	66.86 ± 0.00a	9.60 ± 1.80e‐g	498.10 ± 11.59de
ANOVA						
*F* Storage time	26.81***	21.1***	3.01*	10.9***	5.55***	8.13***
*F* Storage time × Melatonin and MAP	11.16***	22.15***	4.25***	8.34***	4.9***	10.44***

*Note:* Different letters in the same column indicates statistical differences at *p* ≤ 0.05.

Abbreviation: ns, not significant.

* *p* ≤ 0.05 and *p* ≤ 0.001.

*** *p* ≤ 0.05 and *p* ≤ 0.001.

**FIGURE 2:**
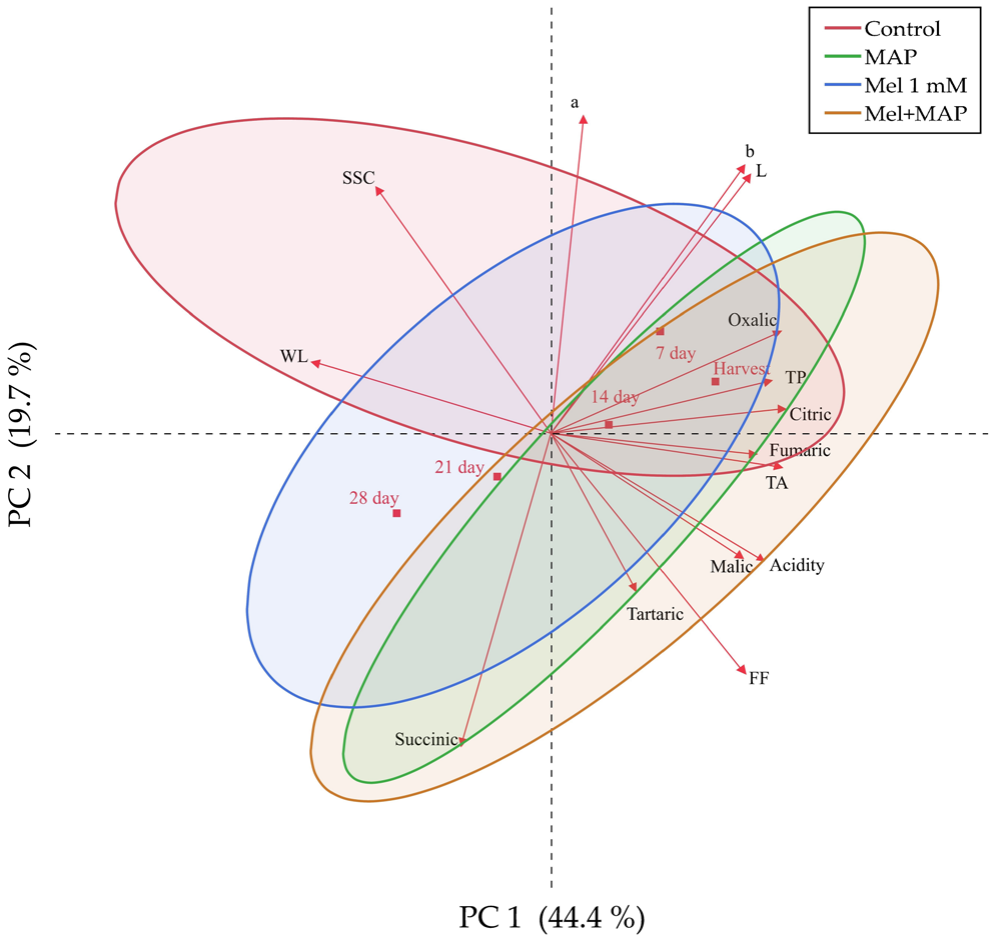
Determination of the variation between post‐harvest treatments, quality traits of fruits and storage duration by PCA.

In this study, heatmap and PCA were performed to statistically reveal the changes in the quality characteristics and organic acid content of persimmon fruits during storage, and to assess the effectiveness of the examined features. The Heatmap analysis showcases the combined evaluation of all treatments and features, while PCA highlights the effectiveness of the examined features within the study. The results of the PCA analysis indicate that the control fruits exhibit a distinctly different trend compared to the treated fruits. The control group fruits exhibited elevated SSC and greater weight loss. In contrast, fruits treated with the combination of melatonin and MAP possess an increased content of organic acids. While melatonin application is less effective at preserving organic acids compared to MAP and the combined treatment, it is still significantly more effective than the control group overall. Heatmap and PCA analyses are widely used in cold storage studies for various fruit types (Küçüker et al. 2024; Kucuker et al. 2023; Yaviç 2024; Taş et al. 2024). The PCA explained 44% of the variance in the features through PC1 and 19.7% through PC2 (Figure 2). In the PCA graph, there is a positive correlation between narrow‐scope features and a negative correlation between broad‐scope features (Garazhian et al. 2020). SSC, weight loss, and a* value exhibited positive correlations with each other, whereas other examined features showed negative correlations. Among organic acids, succinic acid showed a positive correlation with fruit firmness, tartaric acid, malic acid, and acidity content, while other features were negatively correlated. Results from melatonin, MAP, and melatonin + MAP treatments were found to overlap, whereas the control application differed from these treatments. Examining storage durations, at harvest, Days 7, and 14, succinic acid, weight loss, and SSC content exhibited lower values, while other features showed higher values. On Days 21 and 28, succinic acid content was observed to have higher values. Hasan et al. (Hasan et al. 2025) reported similar trends in maintaining SSC, fruit firmness, and reducing weight loss, while they reported enhanced antioxidant enzyme metabolism through the treatment of melatonin combined with MAP. The findings of this study, along with previous research, indicate that the combination of melatonin and MAP provides a comprehensive enhancement of the antioxidant system in persimmons, improving both enzymatic activity and organic acid levels, as well as morphological characteristics.

**FIGURE 3:**
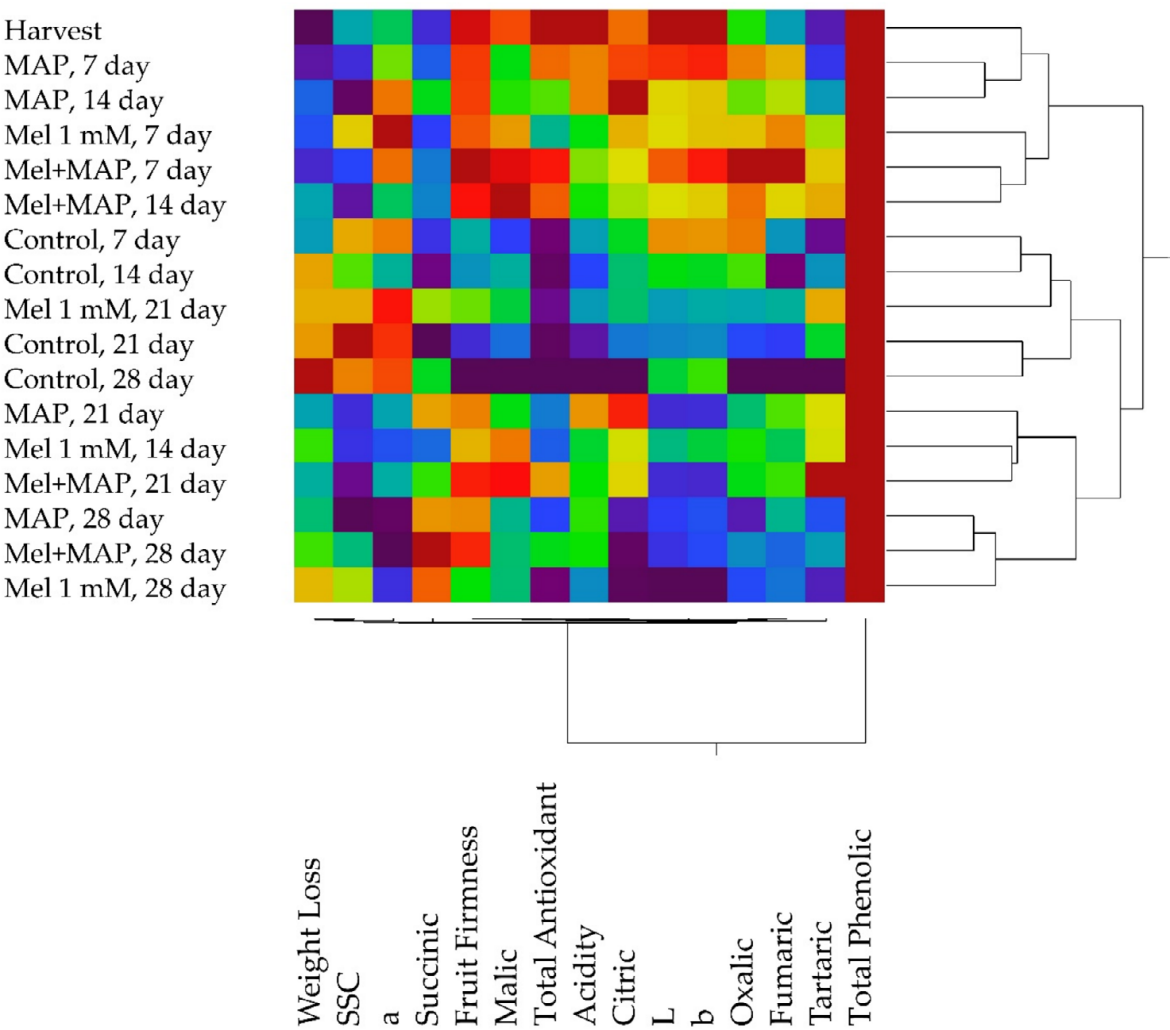
Classification of the relationship between postharvest treatments, storage periods and physicochemical properties of fruits based on Heatmap analysis.

In this study, heatmap analysis was conducted to investigate the interactions between melatonin, MAP, and the combination of melatonin + MAP, as well as their effects on the quality characteristics and organic acid content of persimmon fruits during storage. The analysis indicated that the treatments produced different distributions based on the storage durations. Generally, the combinations of treatments and storage durations were categorized into two main groups. The results from Days 7 to 14 and from the harvest period were grouped together, while the control application and treatments from Days 21 and 28 were placed in a separate category. The features examined were divided into two groups: the total phenolic content of persimmon fruits was categorized separately, while all other features were grouped together (see Figure 3). Heatmap analysis has been extensively used in various storage studies.

**FIGURE 4:**
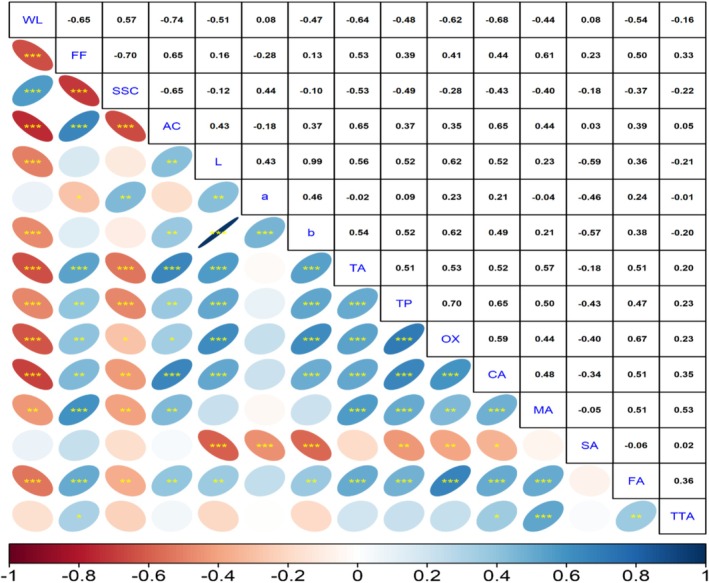
Correlation between quality and biochemical properties of persimmon fruits. The color gradient ranging from red to blue represents correlation values between −1 and + 1. *, **, and *** denote significance levels at *p* ≤ 0.05, *p* ≤ 0.01, and *p* ≤ 0.001, respectively. AC, acidity; CA, citric acid; FA, fumaric acid; FF, fruit firmness; MA, malic acid; OX, oxalic acid; SA, succinic acid; SSC, soluble solid contents; TA, total antioxidant; TP, total phenolic; TTA, tartaric acid; WL, weight loss.

The determination of relationships between the examined characteristics in studies is of great importance for researchers. In this study, significant positive and negative correlations were identified among the examined features. Weight loss, an important quality parameter in storage studies, showed a positive correlation with SSC (*r* = 0.57) and negative correlations with other features. The highest negative correlation was found between weight loss and acidity (*r* = −0.74), and between fruit firmness and SSC (*r* = −0.70). High negative correlations were also identified between weight loss and citric acid (*r* = −0.68), fruit firmness (*r* = −0.65), and total antioxidant activity (*r* = −0.64). The highest positive correlation in the study was observed between L* and b* values (*r* = 0.99). Among the organic acids, no significant correlation was found between succinic acid and total antioxidant activity, malic acid, fumaric acid, and tartaric acid. However, succinic acid exhibited a negative correlation with total antioxidant activity, total phenolic acid, and organic acids. The highest positive correlations were found between oxalic acid and fumaric acid (*r* = 0.67), and oxalic acid and citric acid (*r* = 0.59). High positive correlations were also observed between total phenolic content and oxalic acid (*r* = 0.70) and citric acid (*r* = 0.65) (Figure 4).

## 4 Conclusion

In this study, the effects of melatonin and MAP applications on the quality (weight loss, fruit firmness, SSC, acidity, L*, a*, and b*) and biochemical (total phenolic content, total antioxidant activity, and organic acids) parameters of persimmon fruits were examined. MAP application best preserved the reduction in weight loss, which determines the economic life of the fruit, while melatonin + MAP application effectively slowed down the decline in fruit firmness, which determines the shelf life. Melatonin + MAP application was the most effective method in maintaining the reduction in total antioxidant activity and changes in total phenolic content during storage. MAP application was identified as the most effective method in preserving color changes in fruits during storage. Organic acid content in fruits exhibited changes based on the type of organic acid. Overall, melatonin + MAP and MAP applications were effective in maintaining changes in organic acid content. The results of the study indicate that melatonin + MAP and MAP applications can be used to maintain fruit quality, biochemical, and organic acid content post‐harvest.

## Author Contributions

**Emrah Güler:** formal analysis (equal), writing – original draft (equal), writing – review and editing (equal). **Emine Kucuker:** data curation (equal), investigation (equal), writing – original draft (equal). **Muttalip Gundogdu:** formal analysis (equal), visualization (equal), writing – original draft (equal). **Ahmet Sumbul:** formal analysis (equal), writing – original draft (equal). **Onur Tekin:** writing – original draft (equal). **Bulent Hallac:** writing – original draft (equal).

## Conflicts of Interest

The authors declare no conflicts of interest.

## Supporting information


Table S1.

## Data Availability

The data used to support the findings of this study are included within the article. However, any other information required is available from the corresponding author upon request.
