# Supplementary material for: Preserving Quality and Extending Shelf Life of Climacteric Persimmon Fruits Using Melatonin and Modified Atmosphere Packaging
Source: Food Sci Nutr. 2025 Apr 9;13(4):e70143. doi: 10.1002/fsn3.70143 (PMC11979355; doi:10.1002/fsn3.70143)
Supplement: Supplementary file 1 — Table S1. [file FSN3-13-e70143-s001.docx]

**Sup Table 1**. Effect of melatonin and MAP treatments on quality properties of persimmon fruits during storage.

| Storage time | | WL  (%) | FF  (kg/cm^2^) | SSC  (%) | Acidity  (%) | TA  (µmol 100 TE g^-1^) | TF  (mg GAE 100 g^-1^) |
| --- | --- | --- | --- | --- | --- | --- | --- |
| Harvest | | 000 ± 0.00 | 8.87 ± 0.17a | 15.26 ± 0.24ab | 1.25 ± 0.03a | 31.04 ± 0.56a | 49.48 ± 0.87bc |
| 7^th^ day | | 3.31 ± 0.45c | 8.01 ± 0.37a | 16.42 ± 0.63ab | 0.91 ± 0.04b | 16.94 ± 3.04b | 58.68 ± 2.30b |
| 14^th^ day | | 8.50 ± 1.40b | 7.77 ± 0.53a | 14.61 ± 0.40b | 0.87 ± 0.05bc | 12.97 ± 2.74bc | 74.93 ± 2.08a |
| 21^th^ day | | 10.36 ± 1.74ab | 7.16 ± 0.63a | 17.17 ± 1.05a | 0.83 ± 0.05bc | 9.77 ± 2.50c | 40.66 ± 3.58c |
| 28^th^ day | | 13.11 ± 1.93a | 6.69 ± 0.86a | 16.45 ± 0.77ab | 0.77 ± 0.04c | 6.89 ± 1.77c | 29.23 ± 2.56d |
| Storage time × Melatonin and MAP interaction | |  |  |  |  |  |  |
| Harvest | | 000 ± 0.00 | 8.87 ± 0.17a | 15.26 ± 0.24e-g | 1.25 ± 0.03a | 31.04 ± 0.56a | 49.48 ± 0.87de |
| 7^th^ day | Control | 5.46 ± 0.15d-f | 6.03 ± 0.44bc | 18.77 ± 0.23bc | 0.76 ± 0.03d-f | 4.28 ± 0.03f | 51.45 ± 5.80de |
|  | Mel 1mM | 3.69 ± 0.31ef | 8.48 ± 0.38a | 18.17 ± 0.15b-d | 0.86 ± 0.01c-e | 10.48 ± 0.51d-f | 54.88 ± 2.58c-e |
|  | MAP | 1.66 ± 0.39f | 8.60 ± 0.08a | 14.23 ± 0.20f-h | 1.09 ± 0.04b | 24.40 ± 2.43ab | 61.69 ± 2.17cd |
|  | Mel+MAP | 2.41 ± 0.19ef | 8.93 ± 0.17a | 14.50 ± 0.12f-h | 0.94 ± 0.04c | 28.60 ± 0.00ab | 66.69 ± 1.20bc |
| 14^th^ day | Control | 14.91 ± 2.70b | 5.61 ± 0.26cd | 16.77 ± 0.38de | 0.68 ± 0.05fg | 3.91 ± 0.22f | 64.61 ± 0.64bc |
|  | Mel 1mM | 9.29 ± 0.42d | 8.10 ± 1.31a | 14.30 ± 0.17f-h | 0.84 ± 0.09c-e | 8.02 ± 1.41d-f | 76.69 ± 1.20ab |
|  | MAP | 4.15 ± 0.57ef | 8.58 ± 0.96a | 13.47 ± 0.32h | 1.09 ± 0.08b | 14.84 ± 5.52cd | 77.23 ± 0.84ab |
|  | Mel+MAP | 5.67 ± 1.08d-f | 8.77 ± 0.52a | 13.90 ± 0.06gh | 0.88 ± 0.01cd | 25.11 ± 2.01ab | 81.19 ± 3.90a |
| 21^th^ day | Control | 15.31 ± 4.35b | 3.90 ± 0.06de | 21.97 ± 0.09a | 0.62 ± 0.04gh | 3.78 ± 0.00f | 30.79 ± 2.21g |
|  | Mel 1mM | 14.51 ± 2.44b | 7.70 ± 0.71ab | 18.70 ± 1.20bc | 0.76 ± 0.03d-f | 4.90 ± 0.43ef | 35.24 ± 4.88fg |
|  | MAP | 5.62 ± 0.22d-f | 8.32 ± 0.67a | 14.23 ± 0.20f-h | 1.07 ± 0.06b | 8.78 ± 0.16d-f | 46.24 ± 3.13ef |
|  | Mel+MAP | 5.98 ± 0.30d-f | 8.71 ± 0.61a | 13.77 ± 0.03gh | 0.87 ± 0.01cd | 21.62 ± 6.08bc | 50.38 ± 10.86de |
| 28^th^ day | Control | 22.35 ± 3.27a | 2.33 ± 0.44e | 19.47 ± 1.23b | 0.55 ± 0.03h | 3.53 ± 0.17f | 23.10 ± 2.55g |
|  | Mel 1mM | 14.02 ± 0.47bc | 7.45 ± 1.30a-c | 17.47 ± 0.49cd | 0.75 ± 0.01ef | 4.30 ± 0.22f | 29.43 ± 0.65g |
|  | MAP | 6.63 ± 0.26de | 8.27 ± 0.50a | 13.37 ± 0.29h | 0.89 ± 0.01c | 7.33 ± 1.97d-f | 30.63 ± 1.35g |
|  | Mel+MAP | 9.44 ± 0.29cd | 8.69 ± 1.12a | 15.52 ± 1.06ef | 0.87 ± 0.04cd | 12.40 ± 6.37de | 33.78 ± 10.27fg |
| ANOVA |  |  |  |  |  |  |  |
| F Storage time | | 7.54*** | 1.06^ns^ | 1.74^ns^ | 6.19*** | 51.10*** | 3.89** |
| F Storage time x Melatonin and MAP | | 13.1*** | 7.72*** | 22.67*** | 18.21*** | 12.60*** | 17.28*** |

Different letters in the same column indicates statistical differences at *p*≤0.05. *** indicates *p*≤0.001. ns: not significant.
